# Sustainable Recycling of Waste from Nitrile Gloves: Prolonging the Life Span by Designing Proper Curing Systems

**DOI:** 10.3390/polym14224896

**Published:** 2022-11-13

**Authors:** Nabil Hayeemasae, Abdulhakim Masa, Hazwani Syaza Ahmad, Raa Khimi Shuib, Hanafi Ismail, Indra Surya

**Affiliations:** 1 Research Unit of Advanced Elastomeric Materials and Innovations for BCG Economy (AEMI), Faculty of Science and Technology, Prince of Songkla University, Pattani Campus, Pattani 94000, Thailand; 2 Department of Rubber Technology and Polymer Science, Faculty of Science and Technology, Prince of Songkla University, Pattani Campus, Pattani 94000, Thailand; 3 Rubber Engineering & Technology Program, International College, Prince of Songkla University, Songkhla 90110, Thailand; 4 School of Materials and Mineral Resources Engineering, Universiti Sains Malaysia, Engineering Campus, Nibong Tebal 14300, Malaysia; 5 Department of Chemical Engineering, Faculty of Engineering, Universitas Sumatera Utara, Medan 20155, Indonesia

**Keywords:** natural rubber, nitrile glove, life span, curing system

## Abstract

A massive demand for rubber-based goods, particularly gloves, was sparked by the emergence of the COVID-19 epidemic worldwide. This resulted in thousands of tons of gloves being scrapped due to the constant demand for the items, endangering our environment in a grave way. In this work, we aimed to focus on the utilization of waste nitrile gloves (r-NBR) as a component blended with natural rubber (NR). The life span and other related properties of the blend can be improved by proper control of the chemical recipe. This study assessed three types of crosslinking systems, namely sulfur (S), peroxide (DCP), and mixed sulfur/peroxide (S/DCP) systems. The results indicate that choosing S/DCP strongly affected the tensile strength of the blend, especially at relatively high contents of r-NBR, improving the strength by 40–60% for cases with 25–35 phr of r-NBR. The improvement depended on the crosslink types induced in the blends. It is interesting to highlight that the thermal resistance of the blends was significantly improved by using the S/DCP system. This indicates that the life span of this blend can be prolonged by using a proper curing system. Overall, the S/DCP showed the best results, superior to those with S and DCP crosslinking systems.

## 1. Introduction

The demand for medical and surgical gloves increased significantly with the declaration of the COVID-19 pandemic in March 2020, as demand from traditional users in the medical profession grew and also others began using medical gloves in their daily activities, including front-line workers, transportation workers, and even the general public. Increased global production was only able to accommodate a portion of the increase in demand [1,2]. As a result, the global glove shortage persists and is expected to continue into 2022. There are four main types of gloves that are used in a medical and surgical context, namely natural rubber latex, vinyl, nitrile, and gloves of other materials (e.g., neoprene or polyisoprene). Since entering the market in the early 1990s, nitrile gloves have been the preferred glove type for use in the medical field. Compared to other common medical and surgical gloves (e.g., latex and vinyl), nitrile gloves cause fewer allergic reactions, are more tear-resistant, and are lower priced [3].

During the high demand for nitrile gloves, the products might still be rejected for up to 15% in quality control, due to the strict regulations for nitrile glove products. The largest defects come from latex dipping leaving craters, blisters, and pinholes. Air bubbles and dirt entrapped in the latex compound and a greasy former are the main causes of these defects. Therefore, the glove industries have a major disposal problem in dealing with rejected gloves. Nitrile gloves are similar to other rubber products in being non-degradable material considered a potential feedstock for reclaiming rubber [4,5]. The scrap nitrile gloves are considered waste and are usually simply discarded. However, recycling is one of the best options to solve scrap disposal problems [6]. The utilization of rubber waste has been adopted in several applications within rubber and plastic industries, as well as in construction and building materials and in home decorations [7]. Converting nitrile gloves to a rubber matrix with potential in rubber products is crucial. This can be conducted by blending it with virgin rubber to gain synergistic properties [8]. Blending is also an easy and cost-effective method to produce new combinations of properties. Blends of natural rubber (NR)/recycled nitrile rubber (r-NBR) have been reported previously; however, those studies have had a limited scope, focusing on the compounding and mechanical properties of the blends.

To widen the use of this blend component, other effects must be assessed when seeking the best options to prepare the NR/r-NBR blends. Therefore, it was imperative to study the effects of various alternative vulcanizing systems, namely sulfur vulcanization, peroxide vulcanization, and mixed sulfur/peroxide vulcanization, on the properties of the blends. Generally, the sulfur system is commonly used and is the first choice for curing unsaturated rubbers, as it can provide the rubber vulcanizate excellent mechanical properties superior to those with the peroxide vulcanizing system. However, rubber cured with the sulfur system always has an unpleasant smell due to the elemental sulfur, and the smell induced by the peroxide system is less insulting. The peroxide system also makes the rubber more resistant to elevated temperatures, giving a better compression set and less discoloration of the finished products [9,10,11]. Meanwhile, the drawbacks of this system include a blooming effect, relatively poor mechanical properties, and unwanted by-products after vulcanization. Therefore, proper formulations and processing conditions require a careful selection or tuning, in order to minimize the drawbacks of vulcanizing NR/r-NBR blends.

To date, the attempts to improve the properties of rubber waste blends have involved a third component acting as a compatibilizer, but this adds to the operating costs. However, designing a proper formulation may not increase manufacturing costs. This work assessed the effects of various vulcanizing systems on the curing characteristics, mechanical properties, morphology, and thermal aging properties of NR/r-NBR blends. The point of this interest was not only to gain better durability of the blends but also to increase the life span of the rubber.

## 2. Experimental Details

### 2.1. Materials

**Figure 1:**
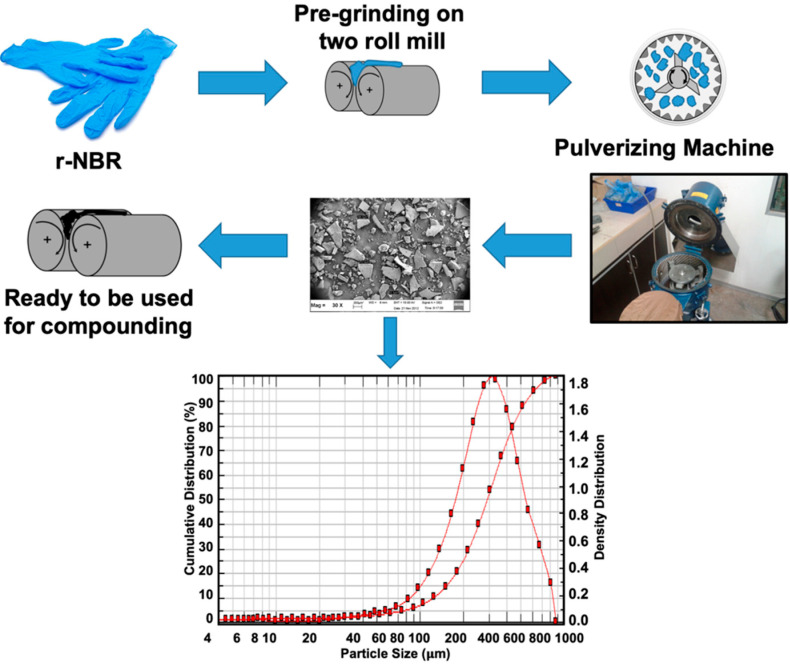
Preparation steps of r-NBR prior to blending with NR.

The main rubber matrices were NR and r-NBR. The SMR L Grade of NR was supplied by Mardec Berhad, Malaysia. The r-NBR obtained from rejected nitrile gloves was supplied by Juara One Resources Sdn. Bhd., Bukit Mertajam, Penang, Malaysia. Figure 1 summarizes the step for preparing r-NBR until it was ready to use. The r-NBR collected from rejected nitrile gloves was pre-grinded by a two-roll mill. This step provided r-NBR with a small size of approximately 1–10 mm. Then, the resultant r-NBR was ground further using a Table Type Pulverizing Machine from Rong Tsong Precision Technology Co., Ltd. (Taichung, Taiwan) to achieve recycled rubber powder. The r-NBR powder was sieved using an Endecott’s siever. The r-NBR was ready to be used as a matrix. Meantime, the particle size of r-NBR was also analyzed by using SYMPATEC HELOS/BF Particle Size Analyzer. It can be seen from Figure 1 that the size distribution of r-NBR is between 125.74 µm to 558.03 µm. The mean particle size of r-NBR is 293.51 μm. The N330 grade carbon black used as a reinforcing filler was purchased from Brenntag Sd. Bhd. (Selangor, Malaysia). Other additives such as an activator (zinc oxide (ZnO) and stearic acid), an accelerator (*N*-cyclohexyl-2-benzothiazolesulfenamide (CBS)), an antidegradant (*N*-isopropyl-*N*’-phenyl-*p*-phenylenediamine (IPPD)), a peroxide (Dicumyl peroxide (DCP)), and a curing agent (sulfur) were supplied by Bayer (M) Ltd., Petaling Jaya, Malaysia.

### 2.2. Preparation of the Blends

The blend formulations are given in Table 1. The point of this study was to minimize the cost of preparing NR/r-NBR blends, so the blends containing one of the three crosslinking systems, namely sulfur (S), peroxide (DCP), and mixed sulfur and peroxide (S/DCP), were compounded on a laboratory-sized two-roll mill (160 mm × 320 mm), model XK-160, following ASTM method D3184. The compounds were then compression molded at 10 MPa each for their respective cure time (t_90_) as determined with MDR 2000.

### 2.3. Measurement of Cure Characteristics

The cure properties of the blends were measured using a Monsanto Moving Die Rheometer (MDR 2000). The respective compounds were tested at a temperature of 150 °C. This was to obtain information regarding the minimum torque (M_L_), maximum torque (M_H_), scorch time (t_s2_), and cure time (t_c90_).

### 2.4. Measurement of Mechanical Properties

Dumbbell-shaped test samples were cut from the molded sheets, prepared according to ASTM D3182. Tensile test was performed according to ASTM D412, at a crosshead speed of 500 mm/min using a universal tester tensile machine (Instron 3366). Hardness measurement was conducted according to ASTM D1415 using a Shore A hardness durometer. The rebound resilience was measured using a Wallace Dunlop Tripsometer according to ASTM D1054. The value of resilience was calculated according to the following equation.

(1) $$Resilience\;\left(\%\right)=\left[\frac{\left(1-cos\theta_{2}\right)}{\left(1-cos\theta_{1}\right)}\right]\times 100$$

where *θ*_1_ is the initial angle (45°) and *θ*_2_ is the maximum rebound angle.

Fatigue tests of rubber blends were run using a Monsanto Fatigue-to-Failure Test (FTFT). The sample was pre-extended to 2.1 times its original length, and it was cyclically stretched at 100 cycle per minute (cpm). Six specimens were mounted for each rubber formulation, and the number of cycles to failure was recorded. The fatigue life in kilocycle was calculated based on the Japanese Industrial Standard (JIS) following the formula below.

(2) JIS Average=0.5A+0.3B+0.1(C+D)

where *A* is the highest value followed by *B*, *C*, and *D* in decreasing order.

### 2.5. Swelling Behavior and Crosslink Density

To measure the crosslink densities of the rubber blends, the swelling uptakes of the rubber blends were determined and correlated with the crosslink density. The swelling uptake was determined by immersing the sheeted NR/r-NBR samples (10 × 10 × 2 mm^3^) in toluene for 168 h (7 days) at ambient temperature. Masses before and after the immersion in toluene gave the swelling uptake and crosslink density. The swelling uptake was calculated as follows.

(3) $$Swelling\;\left(\%\right)=\left(\frac{W_{f}-W_{i}}{W_{i}}\right)\times 100$$

Regarding crosslink density, the masses of the sample before (*W_i_*) and after (*W_f_*) swelling over a period of 72 h were used in the calculation. The cross-link density (v) estimate was based on the modified-Flory-Rehner equation as follows [12].

(4) $$\nu =\frac{1}{{2M}_{c}}$$

(5) Mc=ρ⋅V0⋅(Vr13−Vr2)ln(1−Vr)+Vr+μ⋅Vr2

where *M_c_* is the number-average molecular weight of the rubber chains between crosslinks, *µ* is the rubber-toluene interaction parameter (*µ* = 0.393 and 0.39 for NR-Toluene and r-NBR-Toluene respectively), *ρ* and *V*_0_ are the density of the sample and the molar volume of the toluene (*V*_0_ = 106.2 cm^3^/mol), and finally, *V_r_* is the volume fraction in the swollen specimen. It is defined as follows [13].

(6) $$V_{r}=\frac{\left(D-FT\right)\cdot \rho^{-1}}{\left(D-FT\right)\cdot \rho^{-1}+A_{0}\cdot \rho_{s}^{-1}}$$

where *T* is the mass of the sample, *D* is the mass of the dried sample, *F* is the mass fraction of the insoluble components, *A*_0_ is the mass of the toluene absorbed for the given swelling increment, *ρ* is the density of the sample, and *ρ_s_* is the density of the toluene (0.886 g/cm^3^).

### 2.6. Morphological Studies by Scanning Electron Microscopy

The morphologies of the rubber samples were assessed for the fracture surfaces of tensile samples, using a ZEISS SUPRA^TM^ 35VP scanning electron microscope with GEMINI field emission column. The samples were sputter-coated with gold-palladium before imaging. The point of SEM analysis was to monitor the morphological development of the blends.

### 2.7. Thermo-Oxidative Aging

The heat aging of rubber blends was performed according to ASTM D573. The dumbbell samples were placed in a circulating air oven (Thermolyne-oven series 9000) at 70 °C for 48 h and for 168 h. The heat resistance of a rubber blend was evaluated based on the retention of tensile properties, calculated as follows.

(7) $$Retention\;\left(\%\right)=\frac{Value\;after\;aging}{Value\;before\;aging}\times 100$$

## 3. Results and Discussion

### 3.1. Curing Characteristics

Table 2 lists the minimum (M_L_) and maximum torque (M_H_), and scorch (t_s2_) and cure time (t_c90_), for the NR/r-NBR blends cured using the alternative crosslinking systems. From Table 2, the scorch time for S increased, but the cure time decreased as r-NBR content increased. The scorch safety of the blend improves with an increase in r-NBR content. The presence of crosslink precursors and unreacted curative in the r-NBR will reduce the cure time of the blend. A low concentration of sulfur in the matrix can also decrease the cure time [14,15]. It is noted that S showed the shortest ts_2_ and tc_90_, followed by mixed S/DCP and DCP, in rank order. This observation confirms that the accelerated-sulfur system plays an important role in vulcanizing this blend, while in the peroxide system, the vulcanization time is decided by the half-life of peroxide at a certain temperature. Thus, the vulcanization process is delayed in completing the full decomposition of peroxide. This caused an intermediate vulcanizing time with the S/DCP system, due to the delayed action of the peroxide.

The minimum torque (M_L_) of NR/r-NBR blends for all three systems increased with r-NBR content, while the values were more or less the same across the crosslinking systems. Higher M_L_ was due to an increase in the harder r-NBR phase which increased the viscosity of the compound [16]. The maximum torque (M_H_) in the S system decreased with r-NBR content due to a reduced possibility of sulfur to interact with allylic carbon as NR content was reduced. However, the converse was found for peroxide-containing blends (DCP and S/DCP), and it is clear that peroxide worked well with r-NBR as the M_H_ increased with the addition of r-NBR. Loan [17] reported that the crosslinking efficiencies of peroxide-cured NR and NBR are equal, with one crosslink per molecule. The reason for a higher MH was the tendency of peroxide to interact well in r-NBR, while the sulfur was most likely located in NR. This caused the increase in MH.

### 3.2. Mechanical Properties

**Figure 2:**
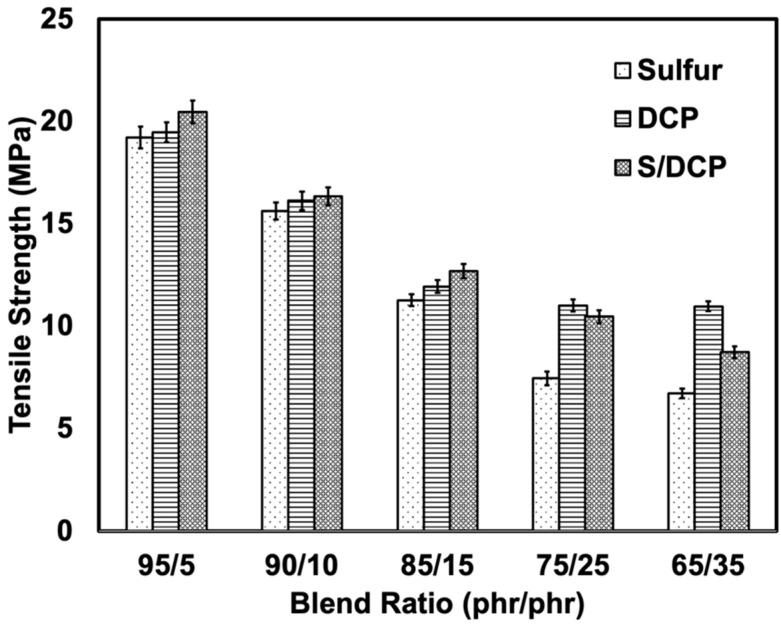
Tensile strength of NR/r-NBR blends prepared with various crosslinking systems.

Figure 2 shows the tensile strengths of the blends cured with different crosslinking systems. The tensile strength decreased gradually with r-NBR content. The reduction of tensile strength was due to the poor compatibility of NR and r-NBR. Another possibility is weak interactions and bonding between the r-NBR particles and NR matrix as the r-NBR was pre-crosslinked. This increased the interfacial tension and deteriorated the strength of the blends. At a fixed blend ratio, the S/DCP exhibited the highest tensile strength, followed by DCP and S systems. This shows a very good compromising effect of peroxide and sulfur, which function well for NR/r-NBR blends. The highest tensile strength was achieved with the S/DCP system might be due to increased crosslink density and stable C-C and sulfidic bonds obtained from peroxide and sulfur [18,19,20]. r-NBR is functioning well with the peroxide, especially at the higher contents of r-NBR (25 and 35 phr of r-NBR), as seen from the tensile strengths.

**Figure 3:**
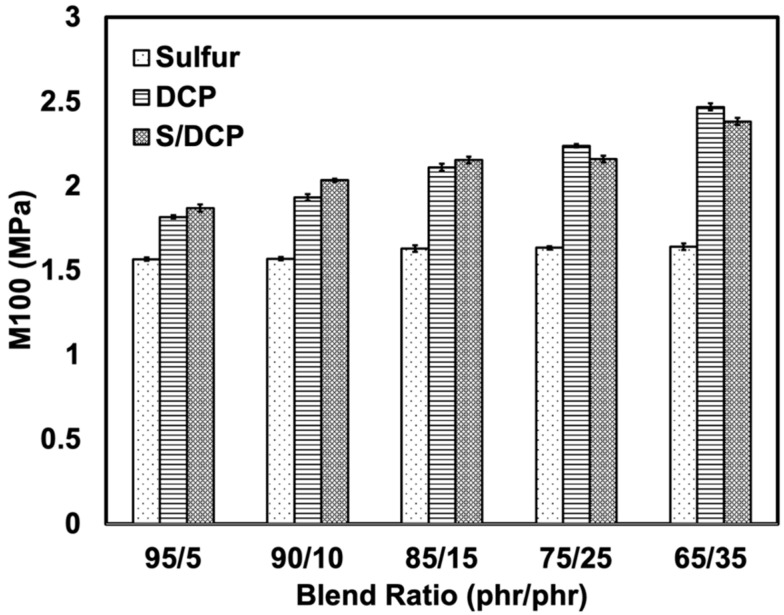
Modulus M100 of the NR/r-NBR blends with various crosslinking systems.

**Figure 4:**
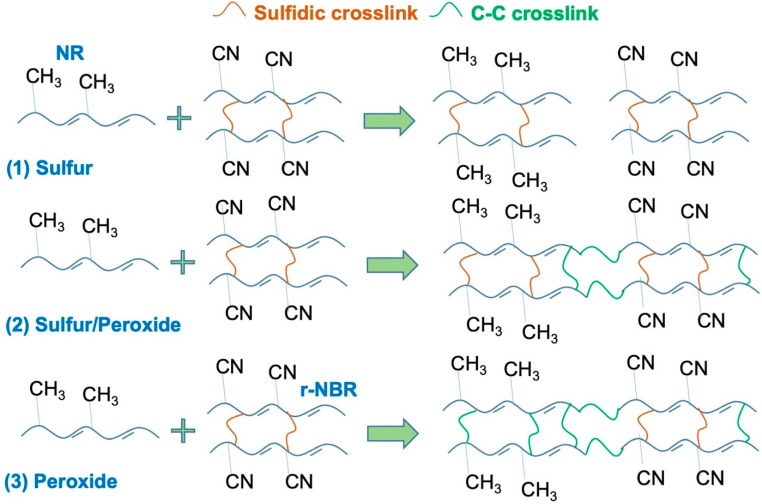
Schematic illustration of crosslinks observed in the blends.

The effects of crosslinking systems on the tensile modulus are shown in Figure 3, for the NR/r-NBR blends. It was reported in terms of stress at 100% strain (M100). The tensile modulus indicates the rigidity of the rubber vulcanizate influenced by the crosslinking or viscoelastic behavior of the rubber vulcanizate. The modulus slightly increased with r-NBR content. This is simply because the r-NBR used in this work was pre-crosslinked, so it was hard and rigid, and therefore it increased the modulus of the blends. At a similar blend ratio, S/DCP gave a higher modulus than DCP and S, because the peroxide provided stable C-C bonds. This can be explained together with schematic reactions presented in Figure 4. In the sulfur-vulcanized system S–S linkages are formed, while in peroxide vulcanizing more stable C–C linkages are formed. In the mixed vulcanized system both S–S and C–C linkages are presented. Normally, rubber samples that have been peroxide-cured are brittle due to the stable C-C bonds, giving the rubber a high modulus [21,22]. However, the sulfur-vulcanized system provides more flexible chains as it performs sulfidic linkages. It does not stiffen the sample, resulting in a reduction of tensile modulus or stiffness of the blends. When sulfur was also used as a curing agent together with peroxide, it provided co-crosslinking in the rubber blends, increasing the overall crosslink density and tensile modulus of the blend.

**Figure 5:**
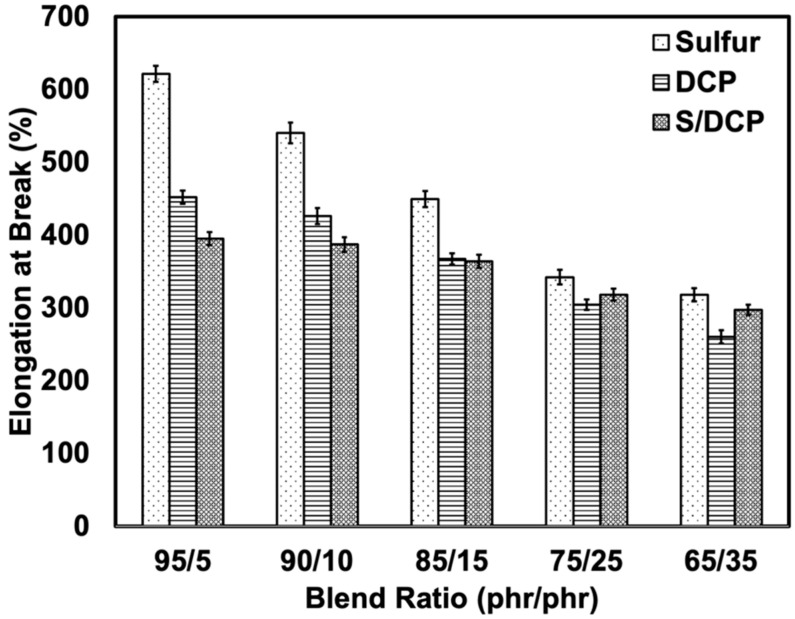
Elongation at break of NR/r-NBR blends with various crosslinking systems.

At the same blend ratio, the mixed system has the highest modulus followed by DCP and S systems in rank order. This is for the blends at relatively high content of r-NBR (25 and 35 phr of r-NBR), where DCP shows a higher modulus than S/DCP. This indicates that peroxide works effectively for r-NBR due to the formation of stable C-C linkages during the vulcanization process [23]. In addition, under applied stress, the C-C linkages did not yield but were more easily broken than the flexible S-S linkages [24]. This made the blends treated with peroxide more brittle than those prepared with sulfur, as can be seen in Figure 5. The elongation at the break of the blends agrees well with the modulus: as the rubber becomes harder it loses flexibility. At the same blend ratio, the sulfur system exhibited the largest elongation at break, followed by DCP and the mixed system. The decrease in elongation at break as r-NBR content was increased was due to the stiffness of the NR/r-NBR blend. The elongation at break depends on the NR content, and on the ability of NR to undergo strain-induced crystallization. The sulfur system can accommodate more stress and exhibits higher elongation due to the highly flexible and labile S-S linkages, which are capable of withstanding high stresses [25].

**Figure 6:**
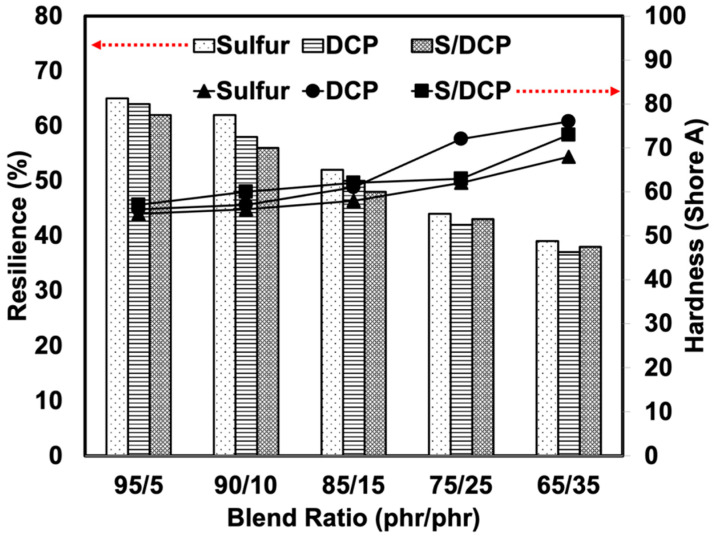
Hardness and resilience of NR/r-NBR blends prepared with various crosslinking systems.

The hardness of the blends also shows the same tendency as the tensile modulus (see Figure 6). This is simply due to the rigidity of rubber vulcanizates. The peroxide system provided the blends with higher crosslink density and elevated stiffness. This caused an increase in the overall hardness of the blends. On comparing the content of r-NBR, more r-NBR made the blend stiffer and reduced the flexibility and elasticity of the rubber blend [26]. The resilience of the blends shows a similar trend to the elongation at break. Resilience represents the ratio of energy given up on recovery from deformation to the energy required to produce the deformation [16]. The molecular mobility of the rubber chains decreased due to the increased stiffness and rigidity of the blend. This observation agrees well with the modulus, hardness, and elongation at the break of the blends [27].

**Figure 7:**
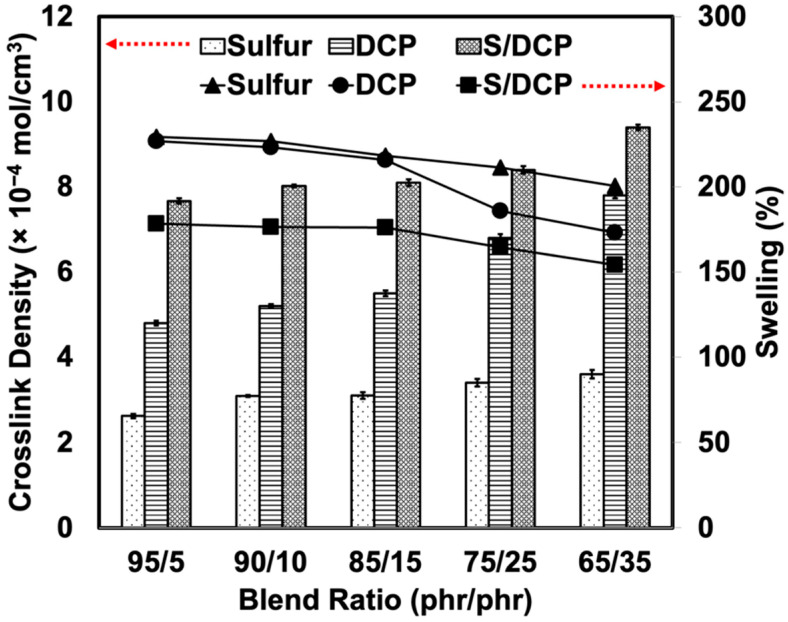
Swelling percentage and crosslink density of NR/r-NBR blends prepared with various crosslinking systems.

To estimate the overall crosslink density in the blends, swelling uptake experiments were carried out. A higher swelling uptake indicates easy solvent penetration to the molecular chains due to less crosslinking [28]. Figure 7 shows the swelling percentages and crosslink densities of the blends prepared with alternative crosslinking systems. The swelling uptake slightly decreased with the addition of r-NBR, while the crosslink density increased as r-NBR content increased. This is simply because the r-NBR was crosslinked before blending, which may reduce the ability of the solvent to penetrate. At a particular blend ratio, the S/DCP system gave the least swelling, while the largest swelling uptake was found when using sulfur. This is expected as the peroxide works effectively in the NR and r-NBR. Stable C-C bonds together with the sulfidic crosslinks enabled higher crosslink density in the blends [29]. This made the rubber swell less when it was prepared with a mixed system.

**Figure 8:**
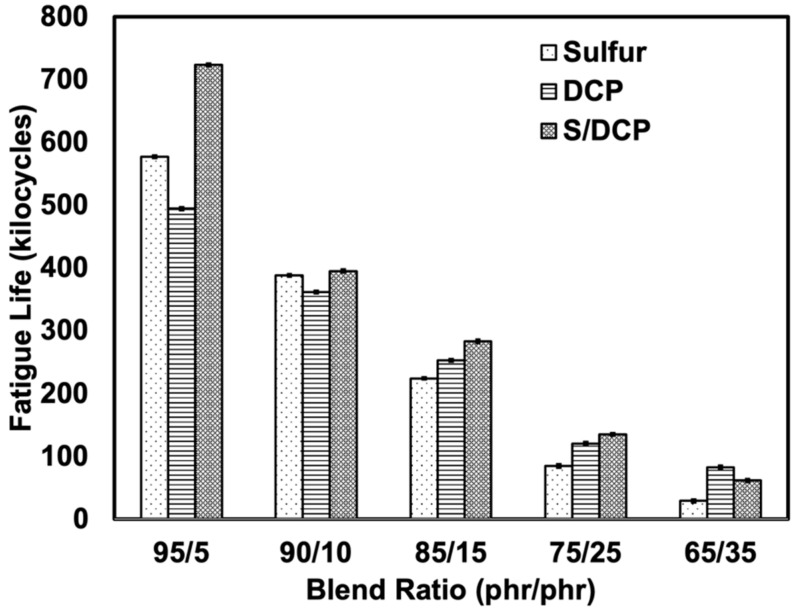
Fatigue life of NR/r-NBR blends with various crosslinking systems.

The fatigue life of the blends was also measured to evaluate the life span of the rubber under cyclic deformations (see Figure 8). The highest fatigue life was obviously found for blends prepared with the mixed system, indicating that the combination of stable C-C bonds and sulfidic crosslinks played a very important role in the dynamic test. The rubber can withstand a longer period of repeating stretching when there is co-crosslinking. However, using peroxide alone did not give a good fatigue life. This may be a limitation of this system: the product will not be appropriate for a dynamic strain application. Moreover, the fatigue life was clearly reduced with the addition of r-NBR, as the rigidity and irregular shapes of r-NBR may be responsible for catastrophic failures of samples subjected to repeating stretching, reducing their fatigue life.

### 3.3. Morphological Studies

**Figure 9:**
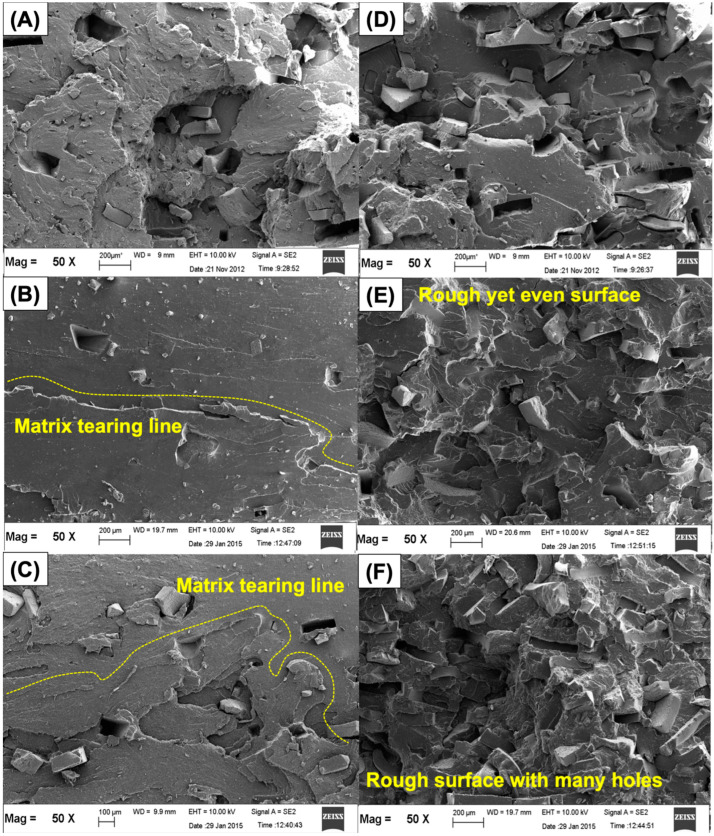
SEM micrographs at 50× magnification of NR/r-NBR blends at 95/5 (phr/phr) cured with S (**A**), DCP (**B**), or S/DCP (**C**); and at 65/35 (phr/phr) cured with S (**D**), DCP (**E**), or S/DCP (**F**).

The morphology of the rubber blends was studied from the fractured surfaces of the tensile test’s specimen. Figure 9A–F show SEM images for the selected blend ratios of 95/5 and 65/35 (phr/phr). On comparing the blend ratios with low and high contents of r-NBR (see Figure 9A–F), homogenous material and fewer holes were observed for the blends containing less r-NBR. This is simply because r-NBR was crosslinked before blending. Its compatibility is poor when blended with a virgin rubber such as NR. One can still see the particles of r-NBR throughout the matrix. This correlates well with the tensile strength observed previously. A higher content of r-NBR decreased the tensile strength. Compared with a particular blend ratio, the sulfur-cured system exhibited many holes and a less homogenous surface than the peroxide or the mixed system. This type of failure indicates that lesser stress caused the catastrophic failure. It is interesting to highlight two different tendencies in the SEM images of peroxide and mixed systems. At a low content of r-NBR (see Figure 9B,C), rougher and uniform surfaces were seen for the mixed system. A reversed observation was found in Figure 9E,F. The image of the peroxide-cured system exhibited a matrix tearing line and a more homogeneous surface rather than the mixed system. This may be the reason why the tensile strength of the blends was found to differ by the content of r-NBR. Peroxide seems to have functioned properly with r-NBR. This image is in good agreement with the tensile strength and other properties observed in the previous sections. The SEM results for the tensile-fractured surfaces are in good agreement with the results obtained by Nabil et al. [28], who studied the tensile-fractured surfaces in relation to variations of vulcanization systems. It was also reported that an increase in energy was responsible for the roughness in matrix tearing at the fractured surface.

### 3.4. Thermo-Oxidative Aging

To assess the efficiency of the blends prepared with various blend ratios, thermal aging was carried out, and the aging resistance of the rubber was evaluated from the retention of mechanical properties after aging. Table 3 and Table 4 list the data on tensile strength, elongation at break, and tensile modulus, before and after aging together with their retention. The tensile strength and elongation at the break of the blends were degraded by thermal aging. This is simply because of the scission of rubber chains when exposed to a long period of heating. Considering the heat resistance of different blend ratios, it was observed that the heat resistance of the blend increased with the addition of r-NBR. There are two main reasons, one being that the r-NBR was crosslinked, and its crosslinking may enhance the heat resistance of the blends, while another reason may be the properties of nitrile rubber itself. Nitrile rubber possesses higher heat resistance than NR, so increasing the content of r-NBR may enhance the heat resistance of the blend. 

As for the tensile modulus (M100), it increased with heat aging. This was probably due to the formation of additional crosslinks. Increased crosslink density after the thermal ageing of the blends was strongly related to the high rate of radical termination in the bulk of the polymer; hence, the material was more cross-linked [28,29]. Comparing different curing systems, the results are in good agreement with the crosslink densities observed in the blends. As expected, the use of a mixed system enabled co-crosslinking, and this has given high retention to the blends. The stable C-C bonds and sulfidic crosslinks are responsible for the enhanced heat aging resistance of the blends. In addition to this, the sulfur system showed the least aging resistance. Sulfidic crosslinks or bonds possess low cleavage energy compared to C-C bonds, and this may be the reason why the least aging resistance was observed for sulfur-cured systems.

## 4. Conclusions

The effects of choice of the vulcanizing system on the curing characteristics, mechanical properties, morphology, and heat aging of NR/r-NBR blends were investigated. The results from this work show that the cure characteristics and mechanical properties of the blends were influenced by DCP and S/DCP crosslink systems. A combination of sulfur and peroxide is more suitable for r-NBR than for NR. This can be seen from the increased tensile strength for blends having a higher content of r-NBR; the increase was 40–60% for such blends. The cure characteristics and mechanical properties of the NR/r-NBR blends depended on the crosslinking agent that was added to the blends. SEM micrographs of the blends agreed well with the tensile strengths observed, and the S/DCP system exhibited the roughest failure surface area indicating that more energy was required in the fracturing. The life span of the blends was considered from the fatigue life and thermal aging of the blends. Fatigue life and thermal resistance were found to be highest for the S/DCP system regardless of the test under dynamic or static conditions. In summary, the blends prepared with a mixed curing system at the blend ratio of 75/25 (phr/phr) of NR/r-NBR provided acceptable overall properties. This approach can be applied to NR/r-NBR blends when high strength and heat aging are of major importance. This formulation is highly suggested to prolong the life span of the blends and is also suitable in applications such as oil seal, gasket, synthetic leather, etc. Such use of r-NBR is one of the best alternatives for making use of waste nitrile gloves that are abundant in the post-COVID-19 era.

## Figures and Tables

**Table 1:** Ingredients used for preparing the blends.

Ingredient	Crosslinking System (phr)		
	Sulfur (S)	Peroxide (DCP)	Mixed (S/DCP)
NR/r-NBR	95/5, 90/10, 85/15, 75/25 and 65/35	95/5, 90/10, 85/15, 75/25 and 65/35	95/5, 90/10, 85/15, 75/25 and 65/35
Zinc oxide	5	5	5
Stearic acid	2	2	2
CBS	1.5	1.5	1.5
IPPD	2	2	2
S	2	-	2
DCP	-	4	4
CB (N330)	30	30	30

**Table 2:** Minimum and maximum torques, and scorch and cure times for the NR/r-NBR blends prepared with various crosslinking systems.

Blend Ratio (phr/phr)	M_L_ (dN.m)			M_H_ (dN.m)			t_s2_ (min)			t_c90_ (min)		
	S	DCP	S/DCP	S	DCP	S/DCP	S	DCP	S/DCP	S	DCP	S/DCP
95/5	0.2	0.2	0.2	11.1	8.9	13.4	1.8	7.3	1.7	5.1	51.4	12.6
90/10	0.2	0.3	0.2	10.7	9.7	13.5	1.8	7.0	1.7	5.0	43.5	12.3
85/15	0.3	0.4	0.2	10.4	9.7	13.6	1.8	6.1	1.7	4.9	41.4	11.7
75/25	0.5	0.6	0.3	9.6	11.3	13.9	1.9	4.8	1.6	4.9	40.9	11.6
65/35	0.7	0.8	0.7	8.8	12.1	15.0	1.9	4.2	1.5	4.4	37.1	10.7

**Table 3:** Heat aging of NR/r-NBR blends prepared with S crosslinking systems.

Property	Sulfur				
	95/5	90/10	85/15	75/25	65/35
Tensile strength					
Before aging (MPa)	19.2 ± 0.5	15.6 ± 0.3	11.3 ± 0.5	7.4 ± 0.6	6.7 ± 0.2
After aging at 48 h (MPa)	7.1 ± 0.5	13.6 ± 0.3	11.5 ± 0.4	7.6 ± 0.4	7.1 ± 0.3
After aging at 168 h (MPa)	6.7 ± 0.4	11.1 ± 0.4	10.3 ± 0.4	6.7 ± 0.4	4.9 ± 0.5
Retention at 48 h (%)	37	87	102	103	105
Retention at 168 h (%)	35	71	91	91	73
Elongation at break					
Before aging (%)	621 ± 4	540 ± 5	449 ± 4	342 ± 3	318 ± 3
After aging at 48 h (%)	279 ± 3	367 ± 3	328 ± 5	274 ± 3	254 ± 4
After aging at 168 h (%)	230 ± 2	243 ± 2	207 ± 6	178 ± 4	188 ± 4
Retention at 48 h (%)	45	68	73	80	80
Retention at 168 h (%)	37	45	46	52	59
M100					
Before aging (MPa)	1.57 ± 0.01	1.57 ± 0.02	1.63 ± 0.02	1.63 ± 0.03	1.64 ± 0.04
After aging at 48 h (MPa)	1.35 ± 0.02	1.41 ± 0.01	1.48 ± 0.02	1.89 ± 0.03	2.95 ± 0.03
After aging at 168 h (MPa)	2.61 ± 0.02	2.65 ± 0.03	2.80 ± 0.03	2.84 ± 0.02	3.10 ± 0.03
Retention at 48 h (%)	86	90	91	116	125
Retention at 168 h (%)	166	169	172	174	189

**Table 4:** Heat aging of NR/r-NBR blends prepared with P and S/DCP crosslinking systems.

Property	DCP				
	95/5	90/10	85/15	75/25	65/35
Tensile strength					
Before aging (MPa)	19.4 ± 0.2	16.1 ± 0.2	11.9 ± 0.4	10.5 ± 0.2	8.7 ± 0.4
After aging at 48 h (MPa)	13.4 ± 0.2	13.0 ± 0.3	11.8 ± 0.4	11.2 ± 0.2	11.2 ± 0.5
After aging at 168 h (MPa)	11.4 ± 0.3	12.9 ± 0.3	10.2 ± 0.3	9.6 ± 0.3	8.4 ± 0.5
Retention at 48 h (%)	69	85	99	107	129
Retention at 168 h (%)	59	81	86	91	96
Elongation at break					
Before aging (%)	452 ± 5	426 ± 3	367 ± 4	318 ± 6	297 ± 5
After aging at 48 h (%)	393 ± 4	383 ± 4	345 ± 4	315 ± 5	297 ± 5
After aging at 168 h (%)	226 ± 3	222 ± 3	195 ± 5	184 ± 5	184 ± 7
Retention at 48 h (%)	87	90	94	99	100
Retention at 168 h (%)	50	52	53	58	62
M100					
Before aging (MPa)	1.82 ± 0.01	1.93 ± 0.02	2.11 ± 0.02	2.15 ± 0.04	2.38 ± 0.02
After aging at 48 h (MPa)	1.51 ± 0.02	1.66 ± 0.03	1.88 ± 0.02	2.60 ± 0.03	3.07 ± 0.02
After aging at 168 h (MPa)	4.44 ± 0.02	4.09 ± 0.04	4.58 ± 0.03	4.71 ± 0.03	5.24 ± 0.03
Retention at 48 h (%)	83	86	89	121	129
Retention at 168 h (%)	214	212	217	219	220
Property	S/DCP				
	95/5	90/10	85/15	75/25	65/35
Tensile strength					
Before aging (MPa)	20.4 ± 0.5	16.3 ± 0.3	12.7 ± 0.7	11.0 ± 0.3	10.9 ± 0.6
After aging at 48 h (MPa)	13.5 ± 0.4	13.0 ± 0.4	13.5 ± 0.5	14.8 ± 0.2	16.7 ± 0.5
After aging at 168 h (MPa)	12.6 ± 0.4	11.7 ± 0.4	13.2 ± 0.6	13.4 ± 0.5	14.8 ± 0.4
Retention at 48 h (%)	66	80	106	135	153
Retention at 168 h (%)	62	72	104	122	136
Elongation at break					
Before aging (%)	395 ± 3	387 ± 2	364 ± 5	304 ± 5	260 ± 4
After aging at 48 h (%)	355 ± 4	348 ± 4	360 ± 6	304 ± 6	268 ± 4
After aging at 168 h (%)	261 ± 3	302 ± 4	295 ± 2	246 ± 4	231 ± 5
Retention at 48 h (%)	90	90	99	100	103
Retention at 168 h (%)	66	78	81	81	89
M100					
Before aging (MPa)	1.87 ± 0.01	2.03 ± 0.03	2.16 ± 0.02	2.24 ± 0.06	2.47 ± 0.01
After aging at 48 h (MPa)	2.99 ± 0.02	3.57 ± 0.04	3.59 ± 0.02	3.81 ± 0.05	4.37 ± 0.03
After aging at 168 h (MPa)	3.95 ± 0.01	4.47 ± 0.02	4.82 ± 0.05	5.17 ± 0.02	5.73 ± 0.03
Retention at 48 h (%)	160	176	166	170	177
Retention at 168 h (%)	211	220	223	231	232

## Data Availability

The data presented in this study are available on request from the corresponding author.
